# The Child Surgical Patient in the Early Twentieth Century

**DOI:** 10.1093/jhmas/jrad005

**Published:** 2023-03-02

**Authors:** Claire Brock

**Affiliations:** University of Leicester, UK

**Keywords:** children, surgery, early twentieth century, patient records, patient voices

## Abstract

In the second half of the nineteenth century, scientific and technological developments in surgery permitted safer procedures to be carried out. Theoretically, therefore, children whose lives would otherwise have been blighted by disease could be saved by timely operative interference. The reality was more complicated, however, as this article shows. Through an exploration of British and American surgical textbooks and an in-depth analysis of the child surgical patient base at one London general hospital, the tensions between the possibilities and the actualities of surgery on children can be examined for the first time. Hearing the child’s voice through case notes allows both a restoration of these complex patients to the history of medicine and a questioning of the wider application of science and technology to working-class bodies, situations, and environments which resist such treatment.

Children in history are often viewed as victims, their voices and actions filtered always through adult perceptions.^1^ If they are heard, as they can be in adult oral history recollections, for example, it is only, as Harry Hendrick has contended, “via whispers and muted articulations.”^2^ The child surgical patient would, of course, be a particularly pertinent example of such silencing and victimisation. In looking back upon the nineteenth-century beginnings of paediatrics, Jonathan Gillis and Patricia Loughran have described the metaphorical encounter between a medical practitioner and a child where the former acts as a veterinarian or an explorer.^3^ However, as this article will show, the reality was much more complicated. Children did tell their own stories, in addition to or in the absence of that of their parents, guardians, or other adult influences. The early twentieth-century Anglo-American hospital population of largely working-class children expressed independent opinions about their health, treatment, and convalescence. In similar fashion to adults, they also narrated their own illnesses, offering assessment of their recently acquired or developing conditions, and reacted - verbally, physically, and both - to treatment. It is vital to acknowledge that the voice of a fourteen-year school child, another of a similar age, newly-employed in their first proper job after leaving education, or even an intelligent eight-year old, were very different from those of an inarticulate baby or toddler. And yet, they are all lumped together, under the assumption that with a child there was an absence of first-person narration; the primary mode of diagnosis had to be through the practitioner’s examination and observation, before a third-person parental history was taken.^4^ In the history of medicine, children are absent presences, operated upon but unsettlingly silent and unconsenting.

Although children were key beneficiaries of nineteenth-century surgical developments, they also complicated the ways in which surgeons operated. Surgery on children was predominantly for temporary conditions (accidents and emergencies) or long-term, chronic illness (such as tuberculosis). Urban working-class children were especially prone to both, given, as we shall see, their propinquity to the effects of industrialisation and related insanitary domestic surroundings.^5^ The longer the condition lasted, the more subject was the child to repeated surgical interventions rather than instant cures. This, in turn, worked against the key contemporary theory that surgery when young for deformities and chronic afflictions gave the child a chance to experience childhood. Too often, chronically-ill children spent months and even years resting or immobile in between procedures. Even then, surgery was inherently conservative and careful. In an age which saw experimentation and a growth in radical procedures, surgery on children was a very different, much more tentative process. By acknowledging the gaps between theory and practice, a more finely grained understanding both of the operative treatment of children and surgical approaches can offer a new perspective on the history of surgery in the early twentieth century.

Patient records, alongside administrative documents, provide a rich but unmined vein of the kind of sources assessed in the recent edited collection *Children’s Voices from the Past* (2019), material which includes artwork, photographs, objects, and spaces, alongside more familiar written sources.^6^ Hospitalised children have been the focus of previous studies, with a centring on the eighteenth century.^7^ Consideration has also been given to the establishment and running of children’s hospitals in the Victorian period, but the general hospital has received much less attention.^8^ Children’s hospitals were strict about the age of admission for patients, and were often smaller and fuller, requiring those seeking treatment to go elsewhere.^9^ A key period for children, the beginning of the twentieth century, has similarly not elicited any in-depth research into the experience of the child surgical patient.^10^ Neither have case notes been explored as material for understanding the historical child’s voice, because they have been deemed to represent “the disease rather than the patient.”^11^ Although it is necessary to understand the limitations of the case note, I disagree with this assessment of the value of these sources. Much can be read and interpreted from the layers of meaning involved in every clinical encounter, and “the patient” (and all their idiosyncrasies) was far more apparent than the sum of their symptoms.^12^ It is important to take into account ethical considerations when accessing patient records which, as with those explored in this article, are open and available on request, if over a century old. With a couple of obvious exceptions, the conditions explored here were neither shameful nor sensitive, but they do contain personal and private information which the (now almost certainly dead) patient has not consented to provide (a double lack with children, of course). The enormous potential of the surgical case note, however, for understanding not only how conditions were treated historically, but also more vitally, the deep social value of exploring access to and the inequalities surrounding health and welfare in the past drives this article’s engagement with these contested sources.^13^ Not only is the hospitalised child separate from its domestic environment, it is also temporarily away from the influence of the family unit. As such, the case note becomes a particularly fruitful source for exploring children’s behaviour.

Through the textbooks written by the earliest British and American specialists, we shall see, firstly, how the child surgical patient was constructed as unique, different, and most vitally, now – theoretically - permanently repairable by those operating in this field. Secondly, turning to patient case notes and examining quotidian life in a metropolitan general hospital in the early twentieth century, the ways in which surgeons dealt with actual child patients can be detailed. The focus here is on London’s Royal Free Hospital, a voluntary institution, as a case study.^14^ All 3000 records between c.1890 and 1914 kept for child patients seen by several of the hospital’s surgeons, James Berry (1860-1946), Joseph Cunning (1872-1948), Henry Work Dodd (1860-1921), and Edmund Roughton (1863-1913) have been consulted.^15^ If hospital patients did not write about their experiences, children were even less likely to reflect on institutional spaces because they were too young or too inarticulate. “Hearing” the child surgical patient in case notes, however, allows the historian to explore reactions and responses to illness, treatment, and convalescence. In so doing, the child, deemed to be silenced by age and responsibility, can be restored to the clinical encounter. A more nuanced understanding of the hospitalised child’s voice, therefore, becomes possible for the first time through close observation of those considered pliant patients. Ironically, it was precisely the new technological methods which permitted safer surgery that caused difficulties for those operating on child surgical patients. More widely, therefore, this article illustrates the chasm between the adoption of improved scientific developments in surgery and their flawed adaptation to an urban hospital population of under-nourished, impoverished bodies, and working-class domestic environments.

## The Child Patient, in Theory

The twentieth century has been labelled by Hugh Cunningham as the “century of the child.”^16^ Indeed, the period between the 1890s and 1914 saw the rights of the child in Britain enshrined in law, and represented the culmination of growing interest in the welfare of the young. By the Children’s Act of 1908, childhood was, as Steven J. Taylor claims, “conceptualised in a modern sense.” At the core of this was the social, cultural, and legal protection of children.^17^ Roger Cooter has additionally noted that between the period of almost half a century between 1880 and the 1920s, child health and welfare became medicalised.^18^ Social and medical concerns about “degeneration” had reached fever pitch on the eve of the Great War.^19^ By 1913, school inspection of approximately 1,830,000 children revealed “an immense amount of physical defect.” Approximate summaries elicited the following: 10% had defective vision; 5% defective hearing; 3% had ear disease; 3% adenoids or enlarged tonsils; half had decaying teeth; 10% had unclean bodies; 1% suffered with ringworm; 2% tuberculosis; 1% heart disease; 10% had malnutrition.^20^ There was an “urgent necessity” to secure and conserve children through the reduction of “defectiveness,” and “all varieties of preventable disorder must be dealt with by effective agencies.” Only through this process could healthy citizens be provided “for coming days.”^21^ Surgery played an important part in this process of “correction.” As Steven King and Steven J. Taylor have remarked, “elective, corrective and reconstructive surgery meant that physical impairment of the sort that might previously have been lived with became increasingly and inexorably remediable.”^22^ This, as we will see, was precisely the way in which surgeons wrote about procedures on children in the late nineteenth and early twentieth centuries.

But it is vital to recognise that the child patient – even in theory – caused surgical anxieties. In 1912, for the American surgeon William Francis Campbell and his co-author, Le Grand Kerr, both the public and the medical profession had come to the recent realisation that “the care of children is a more or less complex problem.”^23^ On the one hand, surgeons could characterise children as “the most appreciative, least complaining, and most trusting patients,” as American cleft palate specialist John Homer Woolsey put it, or as “permit[ting] many liberties to be taken [...] which would otherwise be impossible,” as British surgeon D’Arcy Power remarked.^24^ On the other hand, however, the child, an “unfinished product[…],” required treatment in a “limited space and upon the most delicate structures.”^25^ Children were, as D’Arcy Power’s *The Surgical Diseases of Children* made clear, diametrically opposed, therefore, to the surgeon’s ideal of “big voice, big chest, big merciless hands.”^26^ Operating on children required a diminution of the surgical presence, to acknowledge their alterity. Indeed, nineteenth- and early twentieth-century surgical textbooks described children repeatedly as “difficult,” inherently “peculiar,” or both: “peculiarly difficult.”^27^ Ultimately, they “need and demand more sympathy than any other class of patients.”^28^ In the 1914 second edition of his *Modern Treatise on Pediatric Surgery*, American surgeon Samuel Walter Kelley encapsulated child patients in a single sentence which tussled with their contradictoriness. For Kelley, they displayed “speechlessness, fright, unruliness, lack of comprehension and compliance, and natural restlessness.”^29^ They were both passive and all too active, without speech, yet refused easy examination, uncomprehending and fearful, but physically resistant. Child surgical patients were hard work precisely because of their difference from adults.

One thing that characterised all surgical textbooks focusing on the child, from the earliest onwards, was the benefit to be gained from “modern surgery.” Indeed, surgery on children was more possible precisely because of the developments in contemporary practice. In many ways, children’s “peculiar difficulties” could be distilled or even negated by the surgeon’s newly-acquired scientific aids. Diagnosis and examination would prove far easier through the use of anaesthesia, recommended for the quelling of a restless, squirming child even before any surgery had been considered. Even in the early twentieth century, those dealing with ear, nose, and throat procedures, where children formed a majority of the patient base, could look back with delight at what anaesthesia had enabled. For surgeons in this field, such as the American W.A. Martin, children’s inconsistency made treating them more complex; some were insensible to pain, and did not suffer any nervous disturbance, while others’ nervous shock was severe, terrorised into “naroxysm” by the approach of the surgeon. Anaesthetic changed practice, therefore, and levelled distinctions.^30^ British surgeon John Cooper Forster’s foundational textbook *The Surgical Diseases of Children* appeared in 1860. Forster began with a chapter on anaesthetics, lauding the “great facilities which that agent [chloroform] gives to the operator, and the immense benefit to the patient.” Indeed, its introduction and the corresponding prevention of pain, claimed Forster, had encouraged “rapid strides” to be made in the “manipulative and operative branches” of children’s surgery. So helpful had it been and so safely administered, Forster reported that he now never performed “even the slightest operation without using it.”^31^ Children, as extremely susceptible beings, required little chloroform, if it was good and well administered; they were less sick than adults and recovered consciousness more quickly. Forster had consequently widened its scope beyond surgery itself to include examination, diagnosis, manipulation, dressing, and treatment. It was no wonder that he could extol “the soothing influence” of anaesthesia, given its genuine assistance to the surgeon.^32^ Formerly a tussle between surgeon and patient, anaesthesia removed objections and made the progression of surgery to cure childhood surgical diseases a reality.

Two more key aspects of the “surgical revolution” that were great boons for the children’s surgeon were asepsis and the development of X-ray technology. The former promoted conservatism and a move away from unacceptable, as Campbell and Kerr described it, “surgery in the dark.”^33^ Aseptic methods, indeed, were “particularly beneficent in [their] application to the surgery of childhood.” Carbolic acid, utilised in antiseptic treatment as popularised by Joseph Lister, had been particularly poisonous to children.^34^ British surgeon Edmund Owen added an extra contraindication, that children were “now and then extremely intolerant of the usual antiseptic measures.”^35^ They were prone to accidents leading to fractures, and susceptible to tuberculosis of the bones and joints, so aseptic improvements in the cleanliness of the surgical environment benefited the preservation and protection of youthful limbs, without a need for “dark” radical procedures. Since the late 1890s, X-rays, in turn, allowed pain-free diagnosis and increased precision of bone-setting, which led to the prevention of later deformity from malpositioned fragments. Indeed, children were “attractive subjects for the radiographer,” noted Kelley, because they were small and their bodies readily permeable by the rays.^36^ With anaesthetics, antisepsis, asepsis, and radiography, treatment of injuries such as fractures had become simpler, exact, accurate, and more direct.^37^ Technological improvements in surgical procedures benefited the child patient base in theory because they permitted greater exploration, more precise diagnosis, and most importantly, encouraged safer practise, as well as increased compliance from inherently unruly children.

As textbook analyses recognised, however, alongside the much-improved atmosphere and tools of the operating room, children were still fundamentally difficult patients because they did not “co-operate.” Even with aids to quieten and to keep children immobile, surgeons needed to resort to tricks and stratagems to hoodwink their patients, who did not give their own consent, of course, into compliance. Far from the pliancy and plasticity of childhood, what emerged from textbook explorations was that the theoretical child patient disrupted their own narratives of simplifying pre- and post-surgical processes. In 1912, Campbell and Kerr remarked:

The surgeon must not expect the child to cooperate in the undertaking of a surgical procedure, whether it be an operation, a painful dressing, painful examination, or adjustment of injured parts. Therefore the administration of an anaesthetic is more frequently necessary than in adults. Children bear pain badly, and therefore surgical procedures of even the most ordinary kind are frequently cruel unless the child is protected by an anaesthetic. For instance, in the case of fractures, an accurate adjustment is often impossible without an anaesthetic, and in the matter of diagnosis alone the combined examination under anaesthesia and X-ray is desirable and often absolutely necessary. Even in what are commonly considered the minor surgical conditions, we are convinced that the shock offered to the child by the knowledge of what is taking place more than counterbalances the possible slightly depressing effect of the primary stage of ether anaesthesia.^38^

It was the very nature of the child and the “knowledge” possessed that displaced even the most careful, safe, and scientifically scrupulous procedures. In practice, children were far more attuned to what was going on around them in the surgical environment than theoretically imagined. Surgeons began to realise that anaesthesia often had to be given surreptitiously, rather than applied in the more confident, easy way stated in Forster’s 1860 volume. Owen, for example, recommended that the best method for administering chloroform was while the child was asleep.^39^ In amusingly theatrical language, he also thought it preferable that the anaesthetist appear to the child as the bad man who had hurt them, so that the surgeon could save the day with a remedy to appease the harm inflicted.^40^ Unlike the adult patient, whose trust in the surgeon was paramount to the acceptance of surgical risk by the turn of the twentieth century, the allegedly “trusting” child became apparent only through stratagem and insensibility.^41^ Similarly, the fragility of children’s skin rendered aseptic procedures more complex, and often led to harm; even dressings could “cause most serious sloughing and ulceration” and “give rise to gangrene or to profuse suppuration.” A subcutaneous ether injection could also lead to similar problems.^42^ And of course, children moved, wriggled, and became easily bored, rendering X-raying exceptionally difficult in practice.^43^ In his article on the American public’s reception of the X-ray, Matthew Lavine cites a wonderful example from 1910, which explained how children were fought with fear: “‘I blow a very shrill whistle with considerable force. The noise petrifies the child long enough so that I can make a very good exposure.’”^44^ “[M]erciful aid[s],” as Kelley, citing Owen, remarked, were not always so relieving for patient or surgeon.^45^ The problem was that even with all the advantages of “modern surgery” and “the perfection of technique,”^46^ adaptations and alterations still had to be made for the peculiarities and difficulties of the patient.

What scientific and technological developments allowed in the second half of the nineteenth century, according to those who practised in this area, was more than simply surgery on children. Put succinctly, and this was something on which all agreed: surgery permitted normal life. From the earliest textbooks in the 1860s to the beginning of the twentieth century, children’s surgeons presented themselves as sparing pain and suffering for future adults, saving childhood and essentially protecting children in the way that wider social and cultural awareness of the child’s health and welfare had permitted. As British surgeon Timothy Holmes’s 1868 volume, *The Surgical Treatment of the Diseases of Infancy and Childhood*, put it, “how much may be done by well-timed surgical interference to save life and limb in the affections of childhood.”^47^ The more surgeons developed their understanding of the surgical diseases of childhood, the more experience and confidence in resources would be gained, leading to operations

more in early life than is now the practice, and so [we will] save many children from the painful consequences of growing up malformed during the whole period of childhood. One of the objects of this work is to prove that there are hardly any malformations, which are curable at all, that are not more curable in infancy than in after-life; and further, that far more of these affections are capable of relief from surgical treatment than is generally admitted.^48^

By 1912, Campbell and Kerr had the same positive message, buoyed by exactly that increased specialism indicated by Holmes nearly half-a-century before: “A deformity can often be corrected in early life which later is accompanied by secondary changes which can never be corrected.” “Alertness” to early recognition, diagnosis and treatment could only result in “fewer cripples in the world.”^49^ Theoretically at least, according to textbook discussions, surgery could cure, prevent a stymying of childhood, as well as allow previously moribund children to thrive into adulthood. Surgical disease, warned Campbell and Kerr, was “ever interwoven with problems of nutrition, development, and future efficiency,” necessitating consideration of the whole child.^50^ But, and there was much agreement on this, cures were contingent on early diagnosis and corresponding treatment. Childhood could be saved by surgical means only with the co-operation of the parents or guardians. Congenital malformations, for example, met with in adults were not correctible and “beyond the reach,” as British surgeon Athol A.W. Johnson put it in 1860, “of our art.”^51^ This, of course, meant that the child had to manifest symptoms, the parents notice, and professional advice sought. Essentially, fighting surgical diseases in children was a race against time; once the optimum window for treatment had been passed, deformity or long-term health problems would result. Even with the benefits to be gained from modern surgery, textbook writers recognised that there were still many contingencies as far as the surgical child patient was concerned.

## Children in Surgical Practice

So far we have seen that child patients were both helped by contemporary alterations in surgical practice and also complicated that process through their very fibre as children. Already, without recourse to other sources, textbooks have given a glimpse into the complex ways in which children behaved. This next section will explore actual child cases through patient notes from the Royal Free Hospital between c.1890 and 1914. By doing so, and because of the richness of the sources, it will be possible to ascertain further how children obtained and reacted to surgical treatment. Key to this section will be an interrogation of the child as absent presence in the surgical encounter. Gillis has stated that the “sick child requires a third party to seek help and to bring the problem to the medical system,” the “pediatric clinical encounter is therefore initiated when a sick child is presented to a physician and the physician takes a history from the accompanying adult.”^52^ This narrative was complicated in practice, however, by specifically surgical contexts. Unlike medical treatment, which was often characterised by domestic or patent remedies due to fears over cost, surgical solutions were readily found in voluntary hospitals where fees were not charged.^53^ Indeed, it was this impressive access in the metropolis to a variety of institutional possibilities that led to worries about the abuse of the system.^54^ In the 1890s, Helen Bosanquet was surprised at the remarkable savviness of the would-be patient, who was able to assess the varied treatments on offer and choose where to attend. In spite of contemporary fears surrounding surgical experimentation in hospitals, Bosanquet astutely noted that “the poorer people of London obtain *gratis* medical and surgical treatment of the very first class, and such as none but the very rich can afford to pay for.” Indeed, she continued, “[t]hey are in this respect better off than the classes just above them in the social scale, who can only obtain such medical advice as can be had for a fee.”^55^ This informed searching for health care was an important intervention made by the working classes in the early twentieth-century medical marketplace which has still not been fully explored by historians.

The administrative records of the Royal Free offer a fascinating glimpse into the fact that the seeking of treatment at hospitals was not limited to adults alone. Indeed, in the late 1890s and into the twentieth century, the reports of the Almoner indicated that children were also availing themselves of hospital attention and privileges.^56^ In 1899, over the period of a month, the Almoner recorded 17 children, all under the age of 12, “who came quite alone to the hospital” to seek treatment. Although they were told that they must be brought by a mother or another responsible person next time, in very few cases did they return.^57^ The problem persisted into 1900, as parents were still not accompanying children, nor responding to correspondence from the hospital. Even after home visits, the Almoner concluded that it was not so much a desire to elude enquiry but rather because treatment at a hospital which did not require a subscriber’s letter was so easily obtained.^58^ By 1901, over the period of four months, 21 children had not returned after notes asking parents to accompany them were sent.^59^ The hospital instigated the use of a printed slip, given to the child who had undergone treatment, reminding parents that they must accompany children in order to receive instructions, and that it was not desirable that children should be entrusted with poisonous drugs.^60^ Two years later, the practice was still continuing. In 1903, children were attending alone but the hospital withheld further medicine until a “responsible person” came for it.^61^ Although the numbers remained small – certainly in comparison to the thousands of patients attending each month – that they were ongoing was evidence of concern. Either the parents could not be bothered or were too busy to engage with the necessary waiting for attention, or the children sought treatment without informing or worrying the adults. What was important about this encounter was that it was solely between the child patient and the practitioner: no parents or guardians were involved. The only history obtained was the child’s. While this was clearly a problem for the hospital, they continued seeing and treating solitary child patients, only eventually withholding further treatment until an adult appeared. Even if children were influenced by parental encouragement or, alternatively, by neglect, they were the ones who sought assistance and who, through their own recounting of their symptoms, obtained it. The clinical encounter with children was a very different, more complicated one to that imagined theoretically.

Textbooks described surgery on children as tripartite. As Johnson put it in 1860, childhood surgery was for congenital malformations, accidents, and surgical diseases proper which arose from the previous two or other causes.^62^ Cases seen at the Royal Free followed a similar pattern, with accidents, emergencies, and burns-related injuries forming always over a third and even over half of most surgeons’ yearly child-centred workloads, regardless of their specialist knowledge.^63^ Across all four surgeons considered below, between 1900 and 1914 the average number of emergency patients seen was 44.85% of their annual child patient base. Congenital malformations were surprisingly few, at an average of 8.55% of the surgical load, although Berry’s renown as a cleft palate and hare lip surgeon inflated his cases in this area in comparison to his colleagues. Either “malformation” was accepted at birth, or simply seen by parents and other family members as supportable without correction if it had no effect on quotidian life, especially as children were regularly turned away until they were older for such reasons.^64^ The general category of “Surgical Diseases” vied with accidents and emergencies for most surgeons considered here, at 46.60%, with Dodd and Roughton – ophthalmic and ENT specialists – predictably encountering myriad problems with sight and hearing which took both their averages to over 50% of child patients seen in the early twentieth century. As these statistics show, polarised opposites – acute and chronic conditions - were the experiences of most surgeons working with children between 1900 and 1914.^65^

Surgery on children was predominantly, therefore, either for emergencies or acquired conditions, both exacerbated by early-twentieth century urban society. The rest of this article will explore, in turn, the acute and chronic patients who formed most children on surgical wards. In doing so, case notes will be mined for a dual purpose: to examine the child surgical patients’ active agency in their treatment, as well as understanding more about the experiences children had in hospital. Key to developing a more complex picture of child patients is recognising that they did not always come straight from their homes with parents or guardians. Statistics explain why the hospital had so many problems with injured children turning up alone at “Gate,” the nickname for casualty. In 1911, for example, 173 children were killed in the London streets and 5,075 injured, and the problem was worsening with increasingly mechanised transport and roads tightly crammed with a variety of vehicles. Of the total number of accidents in the metropolis, 34% were child fatalities; three-quarters were boys.^66^ These statistics were reflected in the sheer number of accident cases received at the hospital, which at this point was situated on the busy, central Gray’s Inn Road.^67^ In January 1912, an eleven-year-old boy remarked that this was the third time that he had been run over in seven years. This accident was due to a taxi cab running into the baker’s barrow he was tending as he crossed the road. Previously, at the age of four, he had been knocked down by a carrier’s van, and a year later, by a coal cart; pneumonia, he remarked, giving his own patient history, had set in after discharge from hospital the second time.^68^ As the streets were a respite from cramped homes, it was not surprising that many children brought in after being run over or knocked down were alone.^69^ The most extreme example seen was an unaccompanied four-year old, whose foot was caught by a tram wheel in March 1910, resulting in the need to amputate a toe.^70^ Alternatively, family members only had any contact with the hospital when they came to pick up an injured but recovering child. After a concussion due to a fall in June 1910, nine-year-old Henry Barnes had to wait until his mother came to collect him; she had not yet arrived when he was seen by James Berry and discharged.^71^ In September 1910, an eight-year-old boy, brought into hospital from the street where he had been run over by a taxi cab, was almost scalped, his pericranium divided, and tibia and fibula fractured. Although his mother was contacted and directed to attend, she did not come to the RFH to see her son or to provide any information about him or his medical history. A visit to the family home coincided with her absence.^72^ One four-year-old girl was left alone in hospital in 1911 because her mother “could not be induced to come up.”^73^ Similarly, nobody was with and “no one fetched” seven-year-old Arthur Swain, who claimed he had been run over by a taxi cab. No injuries were found, but he had to stay in hospital until someone turned up to take him home, a day later than arranged.^74^ The number of patients brought alone to hospitals indicated both that the streets were familiar, if dangerous, territory for the metropolitan child, and that they were often part of an outside world which excluded older relations.

Even for accidents within and on the periphery of the domestic confines, very often, and especially in the case of burns, no adult was present to explain the circumstances. In February 1907, three-year old Arthur Jones was brought to hospital by the police, as he had been found completely alone in a room and on fire. He lived for about twelve hours.^75^ There was a similar situation with falls, which took place away from parental observation; windows, railings, and “areas” were the most prevalent means by which children concussed or injured themselves.^76^ After the 1908 Children’s Act, indeed, when the absence of a fireguard leading to the death, burning, or scalding of a child constituted a crime, there may have been an ulterior reason for the absence of anyone over 16. A fine of up to £10 might have been enough to deter presence on the scene.^77^ In November 1908, it was noted that there was no “gate” to prevent the death of one-and-a-half-year-old John Sooby, whose mother had gone to the butcher’s, asking a neighbour to keep an eye on her two children. The neighbour was occupied in the wash-house and had seen the children at the window briefly. Twenty minutes later, when the mother returned, the younger child was on fire; he died the next day.^78^ Two-year-old John F. Smart was luckier when his pinafore caught fire in January 1912. Although he was attended by his grandmother, she was bedridden and the fire was unguarded.^79^ The fascination with the fire was such, however, that even with a fireguard children sometimes ended up in flames. Three-year-old Albert Brown, left alone for five minutes while his mother went to the shops nearby, managed to dislodge an ill-fitting gate and knock the poker out of the fire, setting his clothes alight.^80^ In November 1911, the family of Catherine Spelzini had the requisite guard in front of the fire, but she had simply moved it as soon as she was left alone in the room. Her burns were fortunately superficial.^81^ Heavy, time-consuming family responsibilities such as work, care, and interminable domestic labour necessitating parental absences and reliance on a neighbourly network, made the home a complex site to negotiate for curious or clumsy working-class children alike.

Parents were not always present either when notes were taken.^82^ The “mother’s history” could not be obtained for Florence Wingsore, a nine-year-old girl suffering from suppuration of the frontal sinus. It was the patient herself who remarked that the problem had been apparent for a fortnight, she was in pain from it, and there had been blood when she blew her nose; she had also attended hospital for “fever” approximately two years previously.^83^ An account of one five-year-old girl, admitted with renal calculus, could only be obtained in written form from her aunt.^84^ A seven-year-old girl was readmitted from her home after an accident which left her with fractured femora; she was very dirty and covered in sores and there was no sense of parental presence.^85^ The hospital also admitted a two-year-old boy, who had set himself alight. As his mother was in the infirmary, he had been overlooked at home and his burn was turning septic.^86^ Florence Hunt, aged seven, was also kept on the ward because of “unhealthy surroundings,” and William Tillman was brought in because his mother was occupied with other illness in the house; in addition to scalds, caused by not being given proper attention, he had festering chilblains.^87^ Neglected and ill-looking, nine-year-old Florence Prince was brought to hospital by a “small boy” as she was suffering from a septic foot.^88^ Evidently, for some children, a stay in hospital was a respite from the consequences of impoverished domestic circumstances rather than a trial. Others had been physically or sexually abused at home. For example, five-year-old Elsie Osborne, whose address was given as a barracks, was discovered to have a ruptured hymen, an enlarged vagina, and destroyed tissue separating vagina and anus.^89^ Two of Ethel Vaughan-Sawyer’s patients – Elsie Adams and Violet Donaldson, three and seven respectively – were suffering from gonorrhoea.^90^ The former had been taken to a public lavatory in King’s Cross, but there was no narrative accompanying the latter’s situation. Eleven-year-old Ethel Webb had been attacked on the head by her mother wielding a flat iron, so naturally provided her own history.^91^

Some parents simply did not speak English, and this was especially the case at the RFH as the hospital was very near the metropolitan Italian quarter. In these instances, children were required to narrate their histories, even if they were six, as in the case of Michael Tortora in January 1910. While the notetaker remarked that he was the only one able to explain his situation, so it could not be lucid or reflect upon the past, it was also noted that he spoke English very well and his description was simple, clear, and he placed his accident in time and space.^92^ Michael Tortora’s narrative evidently undermined the notetaker’s initial assumptions. Similarly, children came from orphanages, special schools, and a variety of institutions where their backgrounds were unknown and their family connections obscure. In these instances, the hospital had to ask the child because the institution rarely knew any background. And finally, it is vital to recognise that children attended hospital alone because of embarrassment or because they had been doing something they should not have. This was evidently the case with eleven-year-old James Tillyard, who had injured his scrotum while climbing a wall with spikes on it.^93^ Twelve-year-old George MacDonald had simply sat on a box, but when standing a nail had torn the perineum near his anus; he “came up” alone, his father cited as next of kin.^94^ Similarly, eleven-year-old James Pain was very eager to inform those treating him several times that he had fallen off a woodstack “as high as a house.” His mother had given him medicine, perhaps doubting his tale, and he had walked up alone to hospital.^95^ Brought in by the police, fourteen-year-old clerk Ernest Biggs had shot himself in the head in a suicide bid which eventually succeeded when he died in hospital five days after his admittance. He had been suspected of dishonesty.^96^

While Gillis has concentrated on the ways in which physicians interpreted the mother’s assessment, because she was the one most involved in the clinical encounter and the one so described in paediatric textbooks, many children attended hospital with their fathers.^97^ This was often for practical reasons because fathers were better able to carry or transport an injured child, particularly if they were not babies or very young children, and mothers were occupied with the victim’s siblings or at work themselves. For an extreme example, thirteen-year-old Augustus Reed was collected in a moribund state by his father from a break paid for by the Children’s Country Holiday Fund, where symptoms of appendicitis had been ignored by his hosts; he was brought immediately from King’s Cross station, and died soon after on the operating table.^98^ In some instances, both parents attended and objected to or begged for surgical assistance.^99^ Older children came directly and often alone from their workplaces. Others’ mothers were dead, while stepmothers, grandmothers, aunts, siblings, neighbours, relatives, and friends, as well as school staff, vehicle drivers, policeman, bystanders, and strangers brought children to hospital. A surprisingly large number, given the contemporary belief that the poor abused the hospital system, came straight from a private, fee-receiving doctor. Those who could not afford this possibility went to a chemist or bypassed initial assistance completely, going straight to hospital. Others went to several hospitals before they could be admitted or attended to satisfactorily. One of the more severe examples was five-year-old Henry Ash who in May 1906 had been taken to the Evelina Hospital for Children, St Thomas’ and Great Ormond Street – all without beds – before he was admitted to the Royal Free suffering from empyema, or pus in the pleural cavity.^100^ Before he was seen by Mr. Dodd, after an accident four months previously, Harry Harris had been to the London, Moorfields, the Western Ophthalmic Hospital, and St Mary’s.^101^ Sometimes it was apparent that children had come from a domestic or street setting, but not with whom they had been brought; an indication that the mother’s narrative was neither always sought nor paramount in actuality. What textbooks might have recommended for the taking of patient narratives was not always possible in the realities of the surgical encounter.

While parents, guardians or others could and did explain the child’s condition or history if they were present or cognisant of it, doing so was much more difficult when the senses or less visibly external conditions were involved. Ear, nose, and throat procedures or ophthalmic ones, for example, necessitated an engagement with the patient themselves, no matter how youthful they were. Children necessarily complicated hearing and eyesight tests. Two of Dodd’s three-year-old patients, Augustus Morgan and Leslie Welch, were frustrating because the former’s vision could not be tested accurately because of his age, while the latter was described as “not very reliable.”^102^ But neither could anyone else be asked how well or how poorly they saw objects, so clinical signs as well as narrative, however unreliable, had to be considered for successful treatment. Indeed, it was noticeable that direct quotations from the patient were used more in case notes where the senses were involved. For example, thirteen-year-old Rose Hawkins felt as if her left eye “had a piece of thick muslin in front of it,” while twelve-year-old Alice Smith described her condition powerfully and viscerally, remarking how, in effect, “skin had come over her eyes.”^103^ Four-year-old William Norman felt as if the room was going round and he fell down, the consequence of mastoiditis (the infection of the mastoid bone just behind the ear).^104^ The reason why fourteen-year-old Peek Freans packer Elizabeth Smith could not give a full account of herself, however, was because she had been rendered deaf by a previous attack of scarlet fever. She indicated that headaches plagued her, “to use her own words,” “all over her head.”^105^ Repeated ear problems stemming from mastoid disease caused fourteen-year-old Alice Hanger to complain that “My head’s funny!” The pain and pressure was described as “like a brick.” The specificity of detail caused the notetaker to remark that the patient gave her own history very intelligently, but also later to suggest that her accounts resembled “acting a little.”^106^ Alternatively, patients such as eight-year-old William Hayton, who evidently had hearing problems, could not be tested via the usual ticking watch method because he was so “very unintelligent” and evidently could not respond reliably enough to assess his level of deafness.^107^ In instances such as this, in the absence of personal testimony, nothing was done for the child. When the senses were involved, it was clear that surgeons needed personal engagement and a dynamic with the patient themselves rather than asking others for opinions. Without the relationship between surgeon and child, sensory loss could not be assessed accurately enough for an assured and successful outcome.

**Figure 1:**
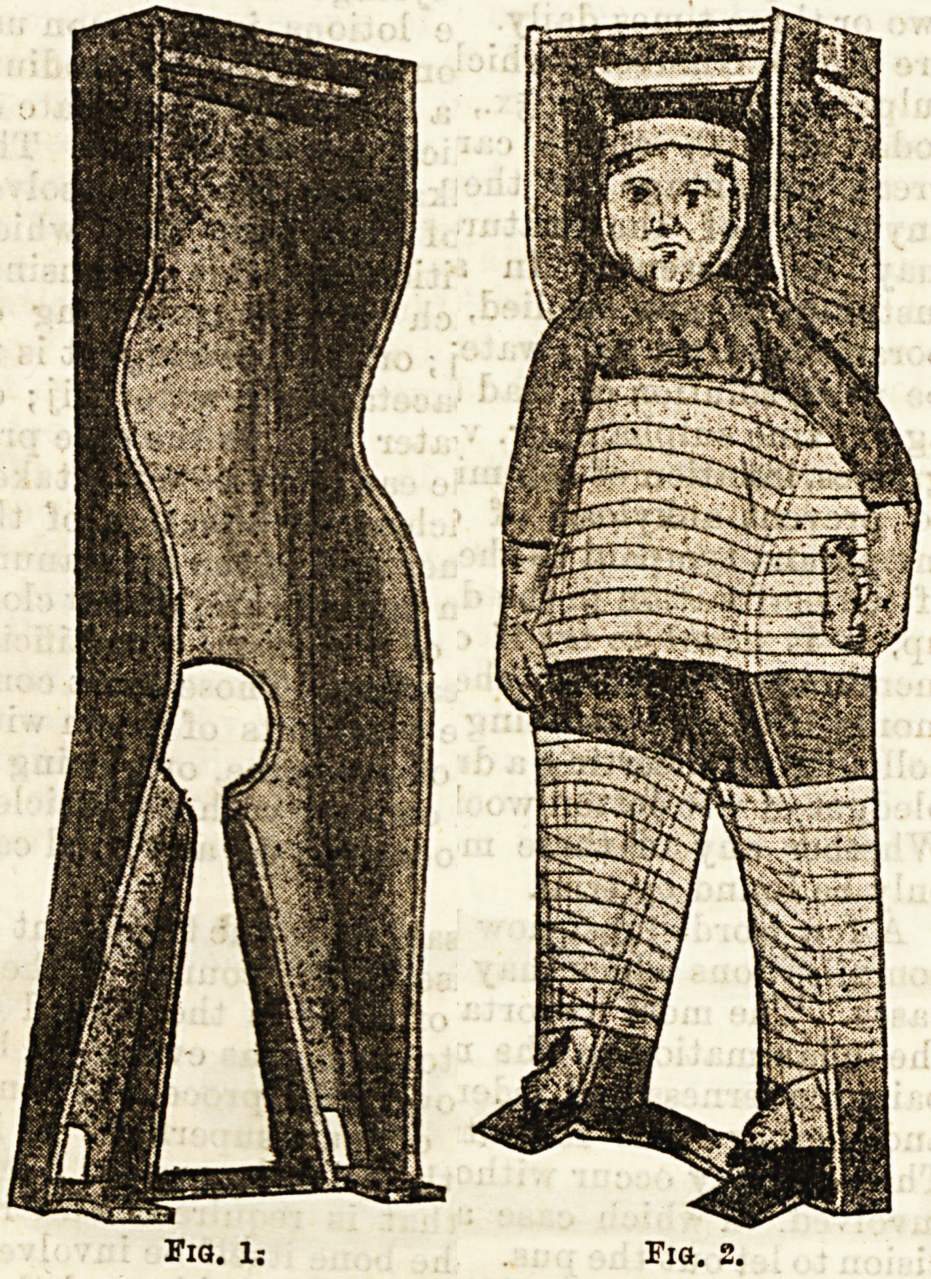
A Phelps Box for Spinal Caries, “The Treatment of Spinal Disease,” *Hospital*, 13 (1892), 153-154; 153. PM1D: 29831749; PMC1D: PMC5251825. Creative Commons 4.0 Attribution 4.0 International License.

As the hospital’s statistics indicated, the number of accidents and emergencies was matched by patients afflicted with chronic “surgical diseases,” who were often transferred from other institutions or convalescent homes, another form of bypassing the parent-surgeon dynamic. Thirteen-year-old Rose Everton, for example, who was suffering from multiple tubercular abscesses even gave her address as a sanatorium where she had been for years.^108^ Patients were regularly moved between the Alexandra Hospital for the Hip, where James Berry also worked, and the RFH for surgical treatment. They also came from a variety of convalescent homes, which very rapidly removed patients from their surroundings if they needed surgical care. Research into convalescence by Eli Osterweil Anders has described the ways in which overcrowded voluntary hospitals found the convalescent home convenient both to free up space and to appease supporters by ensuring the working-class patient could return, fully recovered, to daily productive life.^109^ It is important to recognise, however, that there was not always a one-way trajectory between these institutions. Children were sent from hospital to convalescent home, returned to hospital and then re-released to a convalescent home. This went on for years in some cases, with an occasional return to parents.^110^ That surgical procedures saved childhood was definitely not the case with such patients, moved from institution to institution throughout their younger years. As Cooter has remarked, in the absence of continuity of care, “a vicious downward spiral of ill-health” occurred, as they “drift[ed] in and out of hospitals as acute short-stay cases, rarely achieving lasting cure.”^111^ The problem was that surgery chipped away – quite literally in the case of tubercular joints – but it did not provide an ultimate solution. Too often, with conservative treatment, it could only palliate the condition. Surgical solutions were sometimes mechanical ones, especially in the case of long-term chronic diseases. One of the lengthiest processes was the treatment of spinal caries, where children could spend years immobilised or on their backs. Four-year-old Rose Steele, for example, was recommended a year contained within a Phelps Box (Figure 1).^112^ Improvement could result in the wearing of a variety of plaster jackets – again for years in some cases. Naturally, children grew and became too big for their current jacket, resulting in the need to be replastered and reconcealed within their plaster cages. Despite scientific and technological improvements in surgery, little could be done for these patients and the necessary conservatism of their treatment for active tubercular disease ensured much of their childhood was spent in institutions. Developments in surgical practise did not translate into children’s freedom from pain and suffering.

Alongside emergencies, the impact that suffering from a chronic “surgical disease” had upon the child patient is more than apparent from case notes. And they also make it evident the cost, both in financial and in childhood years, to patient and family. Berry remarked that mechanical means could be purchased for eight years at a cost of £200 – an impossible expenditure for a working-class household - to render Ivor Hobbs’s leg straight. Although he had been spontaneously cured from tuberculosis, without surgical interference, he was now badly deformed.^113^ He remarked that he still felt pain in his knee when the weather was damp, but that nothing had been done for it. When space was at a premium in working-class homes, keeping a child still, clean, and protected was nigh-on impossible. Too often, the relentless need to go to hospital put families off, leading to a deterioration in the condition of the patient. This was complained of numerous times by the hospital’s surgeons, who saw only the neglect and not the difficulties in keeping a sick child clean and safe in unsanitary domestic surroundings.^114^ To cite just a few examples, Emma Cook, five years old and suffering from spinal caries, had been discharged previously from hospital well and able to move her legs. There had been no evidence, lamented Berry, that she had been allowed to rest at home as requested; she was dirty and neglected, incontinent, and unable to walk. Some of this was deliberate, as only when “pinched” and “on pain of punishment” would she flex her limbs. She was kept in hospital for two weeks, mainly to negate the effects of home life.^115^ The parents of Rose Butler had refused operative interference for spinal caries when she was four; now, two years later, her “disease had progressed too far for treatment.”^116^ Mary Dunstan had a congenitally dislocated hip, but at five it was simply too late to operate; Cunning wished she had been seen three years previously. Despite her young age, Mary Dunstan herself was wistful but positive about and accepting of her circumstances. She did not know how long she had been lame, but was not always so; her leg never gave her any pain, and did not wake her up at night. This awareness of her difference, but pride in her similarity, runs counter to other children’s understanding only of the former.^117^ She stated that she was able to run about as fast as other children but became tired sooner.^118^

Although they did not pay for treatment in voluntary hospitals, patients did pay for surgical instruments. Sometimes they were assisted in their purchases by the Almoner, but some could not afford even a small payment. There was “not enough money raised” to buy a spinal jacket needed for seven-year-old “stunted and dwarf-like” James Jeffries, for example, who smiled but maintained enigmatic silence while in the RFH. He had been suffering from spinal caries since the age of two; his parents had also “left off” bringing him to hospital.^119^ After attending St Thomas’ for twelve months, he was “left,” but with his back becoming increasingly curved he was sent to Bart’s, where raw beef and the country were recommended. His parents had not been able to consider the latter, however; after another period of two years, he was brought to the Royal Free, but left without his jacket. Florence Vince was treated in hospital both for burns and for a tuberculous hip initially in June 1908 at the age of two. She spent two weeks on the ward, crying a good deal, while her scalded head was treated, then she was discharged to attend Out Patients and be dressed three times a day; she was readmitted after a few days because of the septic condition of her wound. From July 1908 to January 1909, she remained in hospital and now her hip was treated by extension, bandages, and splints. A Thomas splint was eventually fitted and attached to her chest, thighs, and ankles. This was to be worn for about a year. After convalescence in Sydenham from January to May, she was readmitted, still “very fretful.” The home had an unidentified “outbreak.” After a week with her parents, she was back at the RFH and the splint changed as it no longer fitted her. Now three-and-a-half, she was still in considerable pain, flinching from touch. Her leg was re-extended with weights and tied down to the bed with bandages round her arms. A Thomas splint was reapplied, but it hurt her and created sores; a Liston splint was tried, but required that she “lie more quietly.” By September, she was ready to leave hospital in a new specially-made splint. She was next seen in March 1910, at the age of four. She had not been wearing the splint, which had been “taken off her at home,” and a tuberculous abscess had developed. This was opened up and scraped under anaesthetic, but she scratched at it when no one was looking, resulting in a good deal of bleeding and discharge. The best of surgical intentions could not prevent either individual or family resistance. At the end of the month she was discharged to her parents because she had German measles and was not seen again.^120^

One of the most extreme cases, Emma Barwick, was seen as a child on eight occasions, each time for tuberculosis, between 1904 when she was nine and 1909 when she was fourteen.^121^ As a nine-year old, she reported a history of chronic suffering. She lamented the fact that there had “never been a time” when “her left arm was quite well.” A year before, she had been at Bart’s, where she had been operated upon, but the wound had never healed. In 1900, she had been in the Seaman’s Hospital for surgery on her right thumb, which had also never healed. She had since been at Broadstairs, returning to see Berry, who admitted her. By July 1904, after further surgery, she said her arm no longer hurt her even though a sinus was still apparent, and she was discharged to a convalescent home once more. Her left forearm and thumb were again scraped in January 1905, diseased bone coming away. By March, having been dressed twice a day at Gate, her wounds were quite healed. Two months later, they had reopened; again the radius was gouged out. The same happened in September on her return from another convalescent home where she had been since May. In December, she was back again, to have further gouges from her radius; the same happened in March and May, new sinuses having appeared in her arm while at a convalescent home. Every time, Emma Barwick informed the staff about the by now predictable extent of the ongoing process of healing and inevitable return to discharge. She was in extreme pain, but managed to conceal the presence of a new swelling from Mr. Berry, as she did not feel it was important, a fascinating example of a child assessing her own health through experience. Emma Barwick had also treated the wounds herself at home with Condy’s Fluid. By this point, further surgery was not attempted, and the affected areas were soaked in Wright’s solution and powdered with lead nitrate.

In 1909, at fourteen she was back in hospital having been there “repeatedly,” as her notes remarked. She now also had spinal caries, confirmed by X-ray, in addition to her tuberculous thumb and wrist. Her address was a sanatorium in Midhurst, West Sussex. Her condition was considered “quite good” by Mr. Berry, but nothing further could be done for her except to inject tuberculin and encourage her to lie on her back which, she noted, gave her no pain. There were those who received continuity of care, as Emma Barwick’s five years under Berry made clear, but this did not mean that life was any better for the child afflicted with tuberculosis of the bone. For children such as James Jeffries, Florence Vince and, especially, Emma Barwick, childhood was a round of hospital visits, punctuated by surgical procedures and convalescence. It involved being still and quiet, never going to school or playing with friends, and regularly in considerable pain, if they survived. Although surgical textbooks stressed that it was a battle against time to rescue childhood, some children could not be rescued. It was not the idyll dreamt of by those who envisaged surgery’s impact on a child’s life, and it was no wonder that parents “left off” inflicting restrictive treatment on their children.

Child patients were also immobilised by technology in the hospital, or at least that was the intention. As we have already seen, textbooks extolled the wonders of anaesthesia as an agent of control. Coupled with developments in radiography, children’s injuries and especially joint diseases were pinpointed with more accuracy. It undoubtedly permitted a level of examination difficult to achieve before, and in many ways a more precise and helpful diagnosis. Even if they did not always follow advice, adults could be clearly instructed on what to do, whereas in practice, it took more time and effort to engage a child to achieve the best possible results. Multiple X-rays needed to be taken for some restless children; this could lead to poor treatment rather than improved, as bad exposures meant problems were missed and deformities could result. Eight-year-old Thomas Jones’s separated epiphyses had been located because of an X-ray, which could penetrate beneath his swollen arm. This did not prevent, however, a complete loss of sensation. It was hardly a wonder that he was “very low-spirited.” After other attempts, including triple surgery, electricity, and massage, normal sensation had still not returned when he was discharged for the second time after five months in hospital.^122^ Three-year-old Florence Kirby’s condition could not be confirmed because of blurred pictures.^123^ Fear of the loudness or smell of the equipment was not the only problem experienced by a surgical team and, indeed, children often struggled to do the most basic thing: sit or lie still when asked.

But it was not just new technologies that were utilised to prevent pain or keep children quiet. Frequently, immobilisation was achieved through simple methods, such as tying arms or legs to the bed. As well as treatment for those suffering from tubercular joints, enforced stillness was employed for the naughty, destructive, or simply fidgety, regardless of age. Ten-year-old Edith Holdgate, who had suffered from a tubercular hip for most of her life, was admitted to the RFH from the Alexandra Hospital to have her leg, which was shortened and malformed after years of treatment, straightened. This necessitated her being tied to the bed after several procedures to prevent her twisting her pelvis and adducting her leg.^124^ “Fits of passion” (and suggested epilepsy) were the regular outlet for one nine-year-old, according to his father. Bertie Vyse was in hospital with a dislocated thumb; anaesthetic was employed to reduce the dislocation because it could not be done without as he was too “difficult to manage.” He kicked and fought while his hand was being treated on the ward, ensuring the staff had to tie down his other hand and secure him completely to the bed to prevent “unruliness.” Although anaesthetised and splinted, later he tore off his splint three times and the deformity worsened. The thumb was then plastered, but he struggled and kicked “dreadfully.” Bertie Vyse was ushered, gratefully, out of hospital “in the hope” that the digit was in a better position.^125^ Children were not always passive, trusting recipients of surgical care.

Cocaine or eucaine as local anaesthetising agents were particularly recommended in the early twentieth century. Berry remarked on their value in his own *Manual of Surgical Diagnosis* (1904) for diagnostic purposes or allaying pain or spasm “during the instrumental examination of mucous cavities.”^126^ Dodd found, however, that his child cases complicated the administration of local anaesthesia, rendering it ineffective in some surgical procedures. Thirteen-year-old Rose Hawkins and ten-year-old Charles Watt both needed general anaesthetic because the former could not keep still and the latter was very troublesome.^127^ Cocaine could be a useful tool in the surgeon’s armour, but it was not a sufficient or reliable solution with some children. The same problem occurred with spinal anaesthesia, lauded in the 1910s as a “genuine advantage” as far as both the “psychic and the physical” fears surrounding surgery and eminently suitable for children.^128^ When used for only one patient, thirteen-year-old William Curl, it failed, ensuring he felt the first abdominal incision; he was swiftly given a general anaesthetic.^129^ On the other hand, even in the early twentieth century, there were examples of anaesthetic refusal, either from fear – of the process or the sickness from the aftermath - or stoicism. Textbooks remarked upon boys’ mental and physical strength when they denied that they needed anaesthetic for the pain of surgery or dressing. With cautery processes on tubercular joints, marvelled Samuel Walter Kelley in 1914, “boys of eight or ten” “preferred not to take the anesthetic and bore the rapid application of the cautery bravely.”^130^ But female child patients also refused anaesthetic. Fourteen-year-old Alice Newman, after seven years of surgery for hip disease, had reached a limit and requested that her plaster be reapplied without anaesthetising her again. Her request was granted.^131^ For chronic patients, there was no obfuscating about what would go on in hospital. Babies only knew the present and could not reflect or feel pain, contemporary textbooks claimed, but added that it was advisable to maintain silence with older children about the “ordeal and its results,” and they would not fear or dread an operation or “resultant impairment.”^132^ As the experiences of child surgical patients make clear, surgeons had to do a great deal more than make comfortable and amuse those fully aware of what would happen to them.

Although children did not consent to surgery, they could affect outcomes or delay treatment. Parents may have removed children from hospital when they did not agree to procedures, but children also objected. The most dramatic incident occurred with the escape of a twelve-year-old boy from Dodd’s care in May 1900. After eight days, he had obviously had enough.^133^ Babies or very young children were sent out if they were “refractory” and could not be made “tractable.” Albert Boakes had “a habit” of screaming continually, so Berry would not operate. His notes were updated two months later with the information received by letter that he had died, never having received transformative surgery for his cleft palate.^134^ Silence was a powerful weapon employed by the child suspected of ill health and suffering. It was utilised, for example, to protect parents and siblings from worry and cost, to prevent change occurring to daily routines and environments, or from mimicking the value placed by adults on stiff-upper-lipness.^135^ Orphan eight-year-old Frederick Myers had developed chilblains and sores on his feet but had not complained of it for some time. He was treated at Out Patients for a while, but was admitted because he was not healing fully. His brother noted that he was always very quiet and did not play much.^136^ Four-year-old Henry Padmore, suffering from advanced spinal caries, cried out occasionally when on the ward, but when asked if it hurt him anywhere replied that it did not.^137^ Although in considerable pain from necrosis of the jaw, eight-year-old Walter Cutler bore manipulation “without calling out.”^138^ Going back to school, regardless of increasing suffering, was a frequent occurrence. William Hunter, aged eleven, proud of only having missed one day when the pain in his ear was unbearable, made sure he came to the hospital after school had finished.^139^ Richard Nash provided the most dedicated example of school attendance. The ten-year-old had a fall and was concussed but insisted on attending night school because “he was a bright lad and fond of his lessons.” While there, he was sick and fainted; his father collected him, now unconscious, and took him to a doctor who recommended hospital. His recovery a week later was evident because he sat up in bed and read eagerly.^140^ Fourteen-year-old Catherine Willoughby had finally been admitted for appendicitis after two previous attacks. Initially, she had been so nervous about an operation that she had denied any pain when at University College Hospital. She had been taken to the Royal Free on her second attack, but refused to be examined, leading to a second dismissal. It was interesting that she was operated on “immediately” next time, having finally admitted to severe pain.^141^

On the other hand, over-reactions, naturally very frequent in screaming babies such as Albert Boakes, were also to be seen in older children. Ten-year-old Florence Payne needed to “learn to be tractable,” a process which took nineteen days, before Berry would operate on her. The staff dismissed mental deficiency and found her only hysterical; she was described as excessively excitable, nervous, screaming, crying, and cowering with fright on the very smallest provocation.^142^ In the early twentieth century, a level of maturity and stoicism was required of the child surgical patient, alongside and indeed complementary to technological improvements. Despite the claim that “psychic neurotic influences” were “almost entirely absent” in children, they were very much present in many, and frustrated surgeons.^143^ In spite of all their perceived differences from adults, children proved similar in their reactions to and fears about surgical procedures, as we have seen. Surgeons’ contradictory expectations were evident here; children would not think about disease and its outcomes, but they were also required to rationalise their treatment. Without the “correct” behaviour, character, and attitude for the process, surgery could not be carried out. The expectation that patients behave properly once on the ward, undergoing procedures, and convalescing was as expected for children as it was for adults.

## Conclusion

By considering textbook children alongside their actual counterparts, it has been possible for the first time to place child surgical patients back into the history of medicine. Case notes have permitted the “hearing” of individuals’ voices, a greater understanding of how and why children were in hospital, and a focus on the ways in which they were treated. Surgeons were aided by advances in science and technology, but the child patient also disrupted and affected the most “modern” of treatments. In practice, and a fundamental finding for the history of surgery more generally in this article, is that such theoretical leaps and bounds stuttered when applied to real patients, a difficulty highlighted particularly by children. The patients explored here have leapt from the case notes as individuals narrating their own histories. Six-year-old Mark Keen pronounced kerb as “‘kerve’” and keenly described his previous hospital stay for ‘“Poisoned Blood.’” Robert Buckingham, aged five, called for “‘Mummy’” over and over again when he had been run over. Edward Patrick, eight, remarked “‘Oh my head!’” after his accident. Such examples provide the child surgical patient with a voice, but also allow the historian to interrogate fruitfully and ultimately reposition this most “passive” and “trusting” of patients.^144^ As Robert Buckingham’s “Mummy” was not available, the hospital sent for his schoolboy brother to comfort him, complicating the perception by historians of medicine that the encounter between practitioner and child patient was always mediated by the presence of an adult, a parent or guardian. Indeed, what has been most fascinating is the variety of ways in which children ended up requiring surgical care, and also how their treatment was negotiated rather than passively accepted. Operations could undeniably improve social and financial prospects. For example, routine procedures – for adenoids or hernias - enabled scholarships to be awarded, such as those for eleven-year-olds Harry Bracking or Lily F. Collins.^145^ But if surgery on children was intended to correct and improve, too often it resulted in lengthy periods of rest, with operative interventions when malformations occurred. As surgery on children was inherently conservative, intended to preserve, Cooter’s remark that at best it was “merely a means of stabilising the condition” of those affected physically by disease, at worst “it manufactured cripples,” is apt, as we have seen from the cases at one metropolitan hospital.^146^ What is clear, however, is that surgeons were more optimistic than Cooter allows, believing in an optimum time for treatment when they wrote textbooks extolling the value of surgery specifically on children. In reality, procrastination, unsuccessful previous operations, or the family’s delay for a myriad of reasons in seeking care could blight a child’s future. It is vital to recognise that surgery even for acute cases, with the correspondingly short stay in hospital, could leave children with deafness, paralysis, suppurating operation scars, abscesses, malformed limbs, or the need for ongoing skin grafting or plastic procedures. The most problematic operation at the RFH, for example, was for mastoiditis, which both paralysed facial nerves and led to deafness.^147^ Surgery on children was often support rather than cure, a situation exemplified by the prescribing of mechanical means and conservative procedures to treat the chronically ill.^148^ Ultimately, in theory, childhood could be saved; in practice, modern surgery, for all its developments, tried but struggled with the complexities of the child patient.

## Footnotes

1 Sally Shuttleworth, “Victorian Childhood,” *Journal of Victorian Culture* 9 (2004): 107-113. See also Russell Viner and Janet Golden, “Children’s Experiences of Illness,” in *Companion to Medicine in the Twentieth Century*, ed. Roger Cooter and John Pickstone (London: Routledge, 2000), 575-587.

2 Harry Hendrick, *Children, Childhood and English Society 1880-1990* (Cambridge: Cambridge University Press, 1997), 3.

3 Jonathan Gillis and Patricia Loughlan, “Not Just Small Adults: The Metaphors of Paediatrics,” *Archives of Disease in Childhood* 92 (2007): 946–947.

4 Jonathan Gillis, “The History of the Patient History since 1850,” *Bulletin of the History of Medicine* 80 (2006): 490-512, 510-511. Here, I choose fourteen as the end of childhood - a good number (almost 30%) of the patients of this age were still definitely in school before 1914, even though the leaving age was not raised to this until the Education Act of 1918 (8 and 9 Geo.5, c.39). I have also included those older if they are still in school, or ongoing patients if they were initially treated before this age.

5 For more on how “impairing” conditions in Victorian Britain could be, see “Introduction,” in *Disability and the Victorians: Attitudes, Interventions, Legacies*, ed. Iain Hutchison, Martin Atherton, and Jaipreet Virdi (Manchester: Manchester University Press, 2020), 1-17.

6 Kristine Moruzi, Nell Musgrove, and Carla Pascoe Leahy, ed., *Children’s Voices from the Past: New Historical and Interdisciplinary Perspectives* (Cham: Palgrave Macmillan, 2019), especially chapter 1 by Musgrove, Pascoe Leahy, and Moruzi, “Hearing Children’s Voices: Conceptual and Methodological Challenges,” 1-25. See also Colin Heywood, *A History of Childhood: Children and Childhood in the West from Medieval to Modern Times* (London: Polity, 2001), 6, for historians’ ingenuity in finding sources.

7 See for example, Madeleine Mant, “Children in the London: Inpatient Care in a Voluntary Hospital,” *Medical History* 62 (2018): 295-313; Alysa Levene, Jonathan Reinarz, and Andrew Williams, “Child Patients, Hospitals and the Home in Eighteenth-Century England,” *Family and Community History* 15 (2012): 15-33.

8 For the Victorian period, see Andrea Tanner, “Choice and the Children’s Hospital: Great Ormond Street Hospital Patients and Their Families, 1855-1900,” in *Medicine, Charity and Mutual Aid: The Consumption of Health and Welfare in Britain, c.1550-1950*, ed. Peter Shapeley, Anne Borsay, Jean-Luc Pinol, and Richard Rodger (London: Routledge, 2007), 135-162; Elizabeth M.R. Lomax, *Small and Special: The Development of Hospitals for Children in Victorian Britain* (London: Wellcome Institute for the History of Medicine, 1996). Mant’s article on eighteenth-century “Children in the London” is the exception to the focus on the specialist hospital.

9 See for example, patient Lawrence Warren who, at thirteen, was now too old to attend Great Ormond Street Hospital, despite years of previous treatment there. Mr Berry (Men Part I: December 1911-April 1912), H71/RF/B/02/05/026/001, Royal Free London NHS Foundation Trust, London Metropolitan Archives (LMA).

10 For two exceptions, firstly, where children are considered as a significant part of the Edwardian patient base, see Lynsey T. Cullen, “Patient Records of the Royal Free Hospital, 1902-1912” (PhD thesis, Oxford Brookes University, 2012). Cullen notes that there were not many child surgical patients, however, in the samples she took. A wider exploration, such as that done here, elicits a different reading. Secondly, a short but important analysis with an American focus can be found in Robert J. Touloukian, “Pediatric Surgery Between 1860 and 1900,” *Journal of Pediatric Surgery* 30 (1995): 911-916. See also Jack H.T. Chang and John D. Burrington, “Development of Pediatric Surgery,” *Clio Medica* 11 (1976): 197-201. For later twentieth-century child patient perspectives see Louis Dwyer-Hemmings, “‘A Wicked Operation’? Tonsillectomy in Twentieth-Century Britain,” *Medical History* 62 (2018): 217-241; Emm Barnes, “Between Remission and Cure: Patients, Practitioners and the Transformation of Leukaemia in the Late Twentieth Century,” *Chronic Illness* 3 (2007): 253-264.

11 Viner and Golden, “Children’s Experiences,” 578.

12 For the debate surrounding the value of patient records see Claire Brock, *British Women Surgeons and their Patients, 1860-1918* (Cambridge: Cambridge University Press, 2017), 69-74. An excellent analogy is made by Lykke de la Cour and Geoffrey Raume. Even with a “silent” voice, “actions” and “abilities” of patients such as mentally and physically disabled “Lottie C.,” “speak volumes.” See “Patient Perspectives in Psychiatric Case Files,” in *On the Case: Explorations in Social History*, ed. Franca Iacovetta and Wendy Mitchison (Toronto: University of Toronto, 1998), 242-265, 259. Thanks to one of the *JHMAS* reviewers for drawing my attention to this important collection of essays on a variety of case files.

13 For more on medical records, ethics, and digital dissemination, see Jessica Meyer and Alexa Moncrieff, “Family Not to be Informed? The Ethical Use of Historical Medical Documentation,” in *Patient Voices in Britain, 1840-1948*, ed. Anne Hanley and Jessica Meyer (Manchester: Manchester University Press, 2021), 61-87.

14 Brian Abel-Smith’s summary of these hospitals as ones where “governors gave in cash and doctors gave in kind” encapsulates the British voluntary system of this period. See *The Hospitals 1800-1948: A Study in Social Administration in England and Wales* (London: Heinemann, 1964), 35.

15 Not all records have survived and are patchy for the 1890s. Additionally, there will only be a glance at the children seen by the female surgeons, Florence, Lady Barrett (1867-1945), Mary Scharlieb (1845-1930), and Ethel Vaughan-Sawyer (1868-1949), because their roles in the “Diseases of Women” section meant that they saw very few children.

16 Hugh Cunningham, *Children and Childhood in Western Society Since 1500*, 2nd ed. (London: Routledge, 2014), 171-200.

17 Steven J. Taylor, *Child Insanity in England, 1845-1907* (London: Palgrave Macmillan, 2017), 2.

18 Roger Cooter, ed., *In the Name of the Child: Health and Welfare, 1880-1940* (London: Routledge, 1992), 12.

19 See for example, Jonathan Gillis, “Taking a Medical History in Childhood Illness: Representations of Parents in Pediatric Texts since 1850,” *Bulletin of the History of Medicine* 79 (2005): 393-429, 396; Seth Koven, “Remembering and Dismemberment: Crippled Children, Wounded Soldiers, and the Great War in Great Britain,” *American Historical Review* 99 (1994): 1167-1202.

20 T.N. Kelynack, ed., *Defective Children* (London: John Bale, Sons and Danielsson, Ltd, 1915), 5.

21 Ibid., 6.

22 Steven King and Steven J. Taylor, “‘Imperfect Children’ in Historical Perspective,” *Social History of Medicine* 30 (2017): 718-826, 720.

23 William Francis Campbell and Le Grand Kerr, *The Surgical Diseases of Children* (New York: D. Appleton and Company, 1912), 3.

24 John Homer Woolsey, “Congenital Cleft Lip and Palate,” *California and Western Medicine* 26 (1927): 633-636; D’Arcy Power, *The Surgical Diseases of Children and their Treatment by Modern Methods* (Philadelphia: P. Blakiston & Co., 1898), 1.

25 D’Arcy Power, *Surgical Diseases of Children*, 1; Preface to Campbell and Kerr, *Surgical Diseases of Children*.

26 D’Arcy Power, *Surgical Diseases of Children*, 2.

27 Campbell and Kerr, Preface to *Surgical Diseases of Children*.

28 Ibid., 9.

29 Samuel Walter Kelley, *Surgical Diseases of Children: A Modern Treatise on Pediatric Surgery* (1909), 2nd edition (New York: E.B. Treat & Company, 1914), 25.

30 W.A. Martin, “Anesthesia in Ear, Throat and Nose Operations,” *California State Journal of Medicine* 3 (1905): 359-361, 359.

31 John Cooper Forster, *The Surgical Diseases of Children* (London: Parker, 1860), 1. He does except tonsillectomy from this usage.

32 Forster, *Surgical Diseases of Children*, 2.

33 Campbell and Kerr, *Surgical Diseases of Children*, 98.

34 Ibid., 41. For a recent analysis of antisepsis and the differences between Lister and other surgeons in the fight against wound disease, see Thomas Schlich, “No Time for Statistics: Joseph Lister’s Antisepsis and Types of Knowledge in Nineteenth-Century British Surgery,” *Bulletin of the History of Medicine* 94 (2020): 394-422.

35 Edmund Owen, *The Surgical Diseases of Children*, 3rd ed. (London: Cassell and Company, 1897), 9.

36 Kelley, *Surgical Diseases of Children*, 36.

37 Charles Locke Scudder, *The Treatment of Fractures. With Notes Upon a Few Common Dislocations* (Philadelphia, PA: W.B. Saunders, 1900), 9. It is key to the support these developments have upon surgery that this preface is reproduced in all future editions during the period under discussion in this article. It is only omitted in the eighth edition (1915).

38 Campbell and Kerr, *Surgical Diseases of Children*, 57.

39 Owen, *Surgical Diseases of Children*, 5.

40 Ibid.

41 For growing (adult) patient trust in surgery at this period, see Sally Wilde, “Truth, Trust, and Confidence in Surgery, 1890-1910: Patient Autonomy, Communication and Consent,” *Bulletin of the History of Medicine* 83 (2009): 302-330.

42 Owen, *Surgical Diseases of Children*, 10.

43 For an analysis of experimentation on children utilising technology in the name of child welfare, see Susan E. Lederer, “American Children and Medical Experimenters, 1890-1930,” in *In the Name of the Child*, 96-123.

44 Lewis G. Cole, “Speed Mania,” *American Quarterly of Roentgenology* 2 (1910): 134-135, cited in Matthew Lavine, “The Early Clinical X-Ray in the United States: Patient Experiences and Public Perceptions,” *Journal of the History of Medicine and Allied Sciences* 67 (2011): 587-625, 606.

45 Kelley, *Surgical Diseases of Children*, 40.

46 Campbell and Kerr, Preface to *Surgical Diseases of Children*.

47 Timothy Holmes, Preface to *Surgical Treatment of the Diseases of Infancy and Childhood*, 2nd ed. (London: Longmans, Green, Reader, and Dyer, 1869), iv.

48 Holmes, “Preface to the Second Edition,” *Surgical Treatment of the Diseases of Infancy and Childhood*, vii.

49 Preface to Campbell and Kerr, *Surgical Diseases of Children*.

50 Ibid. These claims run counter to the later date suggested by Cooter – the interwar period – when crippling diseases were believed amenable to treatment and preventable. See Roger Cooter, *Surgery and Society in Peace and War: Orthopaedics and the Organisation of Modern Medicine, 1880-1948* (Basingstoke: Macmillan, 1993), 60.

51 Athol A.W. Johnson, *Lectures on the Surgery of Childhood, Delivered at the Hospital for Sick Children* (London: reprinted from the *British Medical Journal*, 1860).

52 Gillis, “Taking a Medical History,” 393.

53 This is not to suggest that parents did not try to treat surgical problems at home initially, as case notes imply they did frequently, especially as far as burns and scalds were concerned. For domestic medical treatments, see Erin Elizabeth Bramwell, “‘She Used to Doctor Us up Herself’: Patent Medicines, Mothers, and Expertise in Early Twentieth-Century Britain,” *Twentieth Century British History* 31 (2020): 555-578; Siân Pooley, “‘All we parents want is that our children’s health and lives should be regarded’: Child Health and Parental Concern in England, c.1860-1910,” *Social History of Medicine* 23 (2010): 528-548.

54 See for example, Keir Waddington, *Charity and the London Hospitals, 1850-1898* (Woodbridge: Boydell and Brewer, 2000); Keir Waddington, “Unsuitable Cases: The Debate over Outpatient Admissions, the Medical Profession and Late-Victorian London Hospitals,” *Medical History* 42 (1998): 26-46.

55 Mrs Bernard Bosanquet, *Rich and Poor* (1896), 2nd ed. (London: Macmillan and Co., 1908), 36. See also Julie-Marie Strange, *Death, Grief and Poverty in Britain, 1870-1914* (Cambridge: Cambridge University Press, 2007), for working-class attitudes to health and treatment. See also Cullen, “Patient Records.” For a specific analysis of the ways in which children’s hospitals were utilised, see Tanner, “Choice and the Children’s Hospital.”

56 “Report of 7 ½ months’ work to end of September 1899, from January 1 (November 15 1899),” Almoner’s Reports (1895-1913), H71/RF/R/01/001, p.92, Royal Free London NHS Foundation Trust, LMA. The almoner was “a social worker attached to a hospital, attending to the aftercare of patients and originally also to patients’ payments,” *Oxford English Dictionary*. For more on the pioneering work of the Almoner at the Royal Free, see Lynsey T. Cullen, “The First Lady Almoner: The Appointment, Position, and Findings of Miss Mary Stewart at the Royal Free Hospital, 1895–1899,” *Journal of the History of Medicine and Allied Sciences* 68 (2013): 551–582.

57 “Report [...] to end of September 1899,” Almoner’s Reports, 92.

58 “Report of 3mths’ work to March 31 1900 (May 2 1900),” Almoner’s Reports, 101.

59 “Report of 4 ½ months’ work from July 1 to December 31 1900,” Almoner’s Reports, 107.

60 “Report of 4 ½ months’ work from July-December 1901,” Almoner’s Reports, 117.

61 “Report of 5 months’ work from July 1-December 31 1903,” Almoner’s Reports, 142-143.

62 Johnson, Lecture I, in *Lectures on the Surgery of Childhood*, 2-3.

63 Most would be described as “general surgeons.” For example, Dodd was an ophthalmologist, Berry specialised in and become internationally renowned for cleft palate surgery, Roughton dealt with ear, nose and throat cases, especially the former. Cunning’s notes do not begin until 1905; Roughton died in 1912; Dodd’s cases stop in 1913, when he retired; and there are no full records for Berry’s male patients in 1914.

64 Because of this, imperforate anus, for example, was counted as an emergency which required urgent attention. For more on the debate over when to treat children surgically who were suffering from congenital malformations see, Claire Brock, “Surgery, Success, and the Role of the Patient in Cleft Palate Operations, c.1800-1930,” *Isis* 112 (2022): 22-44.

65 A century later little had changed, with the majority of cases for 2004-2005 in ENT and trauma and orthopaedics, although both showed a decline from the mid-1990s. See “Table 2: Change in FCEs 1995-1996 to 2004-2005 by main specialty for children age 0-16 (England),” in *Surgery for Children. Delivering a First Class Service. Report of the Children’s Surgical Forum July 2007* (London: Royal College of Surgeons, 2007), 19.

66 “Introduction: 38-40: Children,” *Report from the Select Committee on Motor Traffic; Together With The Proceedings of the Committee* (London: His Majesty’s Stationery Office, 1913), x. See also Roger Cooter and Bill Luckin, ed., *Accidents in History: Injuries, Fatalities and Social Relations: Historical Orientations in History* (Amsterdam: Rodopi, 1997).

67 The hospital is now in the more leafy and spacious North London surroundings of Belsize Park.

68 Leonard Bayliss, Mr Roughton (Men Part I: January-May 1912), H71/RF/B/02/27/022/001. He knew he had suffered from pneumonia twice, but could not remember the other time, suggesting the impact the accident had made.

69 For the benefits and disadvantages of the streets versus the home for metropolitan children, see Anna Davin, *Growing Up Poor: Home, School and Street in London, 1870-1914* (London: Rivers Oram Press, 1996).

70 George Morris, Mr Roughton (Men Part II: February-June 1910), H71/RF/B/02/27/018/002.

71 Henry Barnes, Mr Berry (Men Part II: June-December 1910), H71/RF/B/02/05/022/002.

72 Edward Rush, Mr Roughton (Men Part III: June-December 1910), H71/RF/B/02/27/018/003.

73 Rose Harman, Mr Berry (Women Part I: January-May 1911), H71/RF/B/02/05/025/001.

74 Arthur Swain, Mr Berry (Men Part II: April-December 1912), H71/RF/B/02/05/026/002.

75 Arthur Jones, Mr Roughton (Men Part I: January-February 1907), H71/RF/B/02/27/012/001.

76 “Areas” were sunken courts giving access to the basement of a house, which were separated from the pavement by railings, and which were accessed via a flight of steps, *Oxford English Dictionary*.

77 15: “Exposing children to risk of burning,” Part II: Prevention of Cruelty to Children and Young Persons, Children’s Act, 1908 [8 Edw. 7, Ch.67], 14. See also Vicky Holmes, “Absent Fireguards and Burnt Children: Coroners and the Development of Clause 15 of the Children Act 1908,” *Law, Crime and History* 2 (2012): 21-58.

78 John Sooby, Mr Roughton (Men Part II: April-December 1908), H71/RF/B/02/27/014/002.

79 John F. Smart, Mr Roughton (Men Part I: January-May 1912), H71/RF/B/02/27/022/001.

80 Albert Brown, Mr Roughton (Men: Part II: February-July 1906), H71/RF/B/02/27/010/002.

81 Catherine Spelzini, Mr Roughton (Women Part II: June-December 1911), H71/RF/B/02/27/021/002.

82 Gillis suggests parents are “inevitably ‘present’ even when they are not mentioned” in medical texts, “Taking a Medical History in Childhood,” 394.

83 Florence Wingsore, Mr Roughton (Men and Women: January-November 1904), H71/RF/B/02/27/007.

84 Primrose Hill, Mr Berry (Women Part I: January-June 1909), H71/RF/B/02/27/021/001.

85 Florence Lewis, first stay in Mr Roughton (Women Part II: June-December 1906), H71/RF/B/02/27/011/02; readmittance misfiled in Mr Roughton (Men Part III: July-December 1906), H71/RF/B/02/27/010/003.

86 Harry Lyall, Mr Cunning (Men and Women Part I: May 1911-August 1912), H71/RF/B/02/12/003/001.

87 Florence Hunt, Mr Berry (Women Part II: June-December 1910), H71/RF/B/02/05/023/002; William Tillman, Mr Roughton (Men Part II: February-July 1906), H71/RF/B/02/27/010/003.

88 Florence Prince, Mr Berry (Women Part I: January-May 1913), H71/RF/B/02/05/029/001.

89 Elsie Osborne, Mr Berry (Women Part II: July-November 1901), H71/RF/B/02/05/005/002.

90 Elsie Adams, Ethel Vaughan-Sawyer (Women Part II: June-December 1912), H71/RF/B/02/31/005/002; Violet Donaldson, Ethel Vaughan-Sawyer (Women Part I: January-May 1911), H71/RF/B/02/31/004/001.

91 Ethel Webb, Mr Berry (Women Part I: January-June 1907), H71/RF/B/02/05/017/001.

92 Michael Tortora, Mr Berry (Men Part I: December 1909-May 1910), H71/RF/B/02/05/022/001.

93 James Tillyard, Mr Berry (Men Part I: June 1899-June 1900), H71/RF/B/02/05/002/001.

94 George MacDonald, Mr Cunning (Men Part I: January-June 1914), H71/RF/B/02/12/004/001.

95 James Pain, Mr Roughton (Men Part I: December 1907-May 1908), H71/RF/B/02/27/014/001.

96 Ernest Biggs, Mr Roughton (Men Part I: December 1910-June 1911), H71/RF/B/02/27/020/001. This case made the national press, which reflected on the boy’s melodramatic gestures before he committed the deed.

97 Gillis, “Taking a Medical History.”

98 Augustus Reed, Mr Roughton (Men Part I: 1901-1903), H71/RF/B/02/27/005/001.

99 Objections were loudly raised when eyes were involved. The parents of Harry Davies, for example, both refused surgery, Mr Dodd (Men and Women Part II: 1897-1903), H71/RF/B/02/13/001/002. Isaac Grosman’s parents removed him before treatment could be carried out, Mr Dodd (Men and Women: January 1910-June 1913), H71/RF/B/02/13/004. On the other hand, compare with John Storey, whose parents were anxious that surgery should be performed in spite of the risk of their child dying during the procedure, Mr Dodd (Men and Women: January 1904-December 1906), H71/RF/B/02/13/002.

100 Henry Ash, Mr Roughton (Men Part II: February-July 1906), H71/RF/B/02/27/010/002.

101 Harry Harris, Mr Dodd (Men and Women: January 1904-December 1906), H71/RF/B/02/13/002.

102 Augustus Morgan and Leslie Welch (the latter twice), Mr Dodd (Men and Women: 1907-1909), H71/RF/B/02/13/003.

103 Rose Hawkins, four visits, and Alice Smith, both Mr Dodd (Men and Women: Part III: 1897-1903), H71/RF/B/02/13/001/003.

104 William Norman, Mr Roughton (Men Part II: February-July 1906), H71/RF/B/02/27/010/002.

105 Elizabeth Smith, Mr Roughton (Women Part II: May-December 1908), H71/RF/B/02/27/015/002.

106 Alice Hanger, twice, Mr Berry (Women Part III: July-December 1902), H71/RF/B/02/05/007/003.

107 William Hayton, Mr Roughton (Men Part III: June-December 1910), H71/RF/B/02/27/018/003.

108 Rose Everton, Mr Cunning (Women: January-December 1914), H71/RF/B/02/12/005.

109 Eli Osterweil Anders, “‘So delightful a temporary home’: The Material Culture of Domesticity in Late Nineteenth-century English Convalescent Institutions,” *Journal of the History of Medicine and Allied Sciences* 76 (2021): 264-293.

110 For more on the “contested” care of children with disabilities, see Anne Borsay and Pamela Dale, ed., *Disabled Children: Contested Caring 1850-1979* (London: Pickering and Chatto, 2012).

111 Cooter, *Surgery and Society*, 62.

112 Rose Steele, Mr Berry (Women Part II: June-December 1905), H71/RF/B/02/05/015/002.

113 Ivor Hobbs, Mr Roughton (Men Part I: January-May 1912), H71/RF/B/02/27/022/001. With the parliamentary debate around the “minimum wage” suggesting 30s a week in 1913, an expenditure of £25 a year for nearly a decade on surgical appliances could simply not have been borne. See for example, “Minimum Living Wage,” *Hansard* 50 (13 March 1913): cc.458-573.

114 For a lively, oral history account of the difficulties in dealing with splints, high boots, and crutches in a metropolitan working-class Edwardian home, see “Thomas Morgan” in Thea Thompson, *Edwardian Childhoods* (London: Routledge, 1981), 9-35, 26.

115 Emma Cook, Mr Berry (Women Part I: January-June 1904; and Part II: June-December 1904), H71/RF/B/02/05/010/001-002.

116 Rose Butler, Mr Roughton (Women: January-November 1909), H71/RF/B/02/27/017.

117 Siân Pooley, “Child Philanthropy, Family Care, and Young Bodies in Britain, 1876-1914,” in *Children’s Experiences of Welfare in Modern Britain*, ed. Siân Pooley and Jonathan Taylor (London: Institute of Historical Research/ University of London Press, 2021), 99-122, 118.

118 Mary Dunstan, Mr Roughton (Women Part I: January-May 1912), H71/RF/B/02/27/023/01.

119 James Jeffries, Mr Berry (Men Part II: June-December 1900), H71/RF/B/02/05/002/002. His father indicated that this was unusual for a very bright and talkative child at home; the notetaker found his smile “rather vacant.” This has a cross next to it in the margin of the note, but remained.

120 Florence Vince, Mr Roughton (Women: January-November 1909) and (Part I: December 1909-May 1910), H71/RF/B/02/27/018-019/001. Originally admitted 22 June 1908.

121 Emma Barwick, Mr Berry (Women Part II: June-December 1904); (Part I: January-June 1905); twice (Part II: June-December 1905); (Part I: December 1905-June 1906); twice (Part I: January-June 1907); (Part I: January-June 1909), H71/RF/B/02/05/011/002; H71/RF/B/02/05/013/001-002; H71/RF/B/02/05/015/001; H71/RF/B/02/05/017/001; H71/RF/B/02/05/021/001. The 1911 Census suggests that Emma Barwick was still alive and living in East Ham, but death records indicated that she was back at Midhurst in 1915, where she died at the age of twenty.

122 Thomas Jones, Mr Roughton (Men Part II: April-August 1905) and (Men Part I: December 1905-March 1906), H71/RF/B/02/27/008/002; H71/RF/B/02/27/010/002.

123 Florence Kirby, Mr Roughton (Women Part II: June-December 1911), H71/RF/B/02/27/021/002.

124 Edith Holdgate, Mr Berry (Women Part I: January-June 1901), H71/RF/B/02/05/005/001.

125 Bertie Vyse, Mr Roughton (Men Part I: 1901-1903), H71/RF/B/02/27/005/001.

126 James Berry, *A Manual of Surgical Diagnosis* (London: J. & A. Churchill, 1904), 35.

127 Both Mr Dodd: Rose Hawkins (Men and Women: Part III: 1897-1903), H71/RF/B/02/13/001/003; Charles Watt, (Men and Women: 1910-1913), H71/RF/B/02/13/004.

128 J.T.J. Morrison, “The Ingleby Lecture on Spinal Anaesthesia by Tropacocaine; with a Review of 1,295 Cases,” *British Medical Journal* 1 (1913): 1305-1313, 1309.

129 William Curl, Mr Roughton (Men Part II: June-November 1909), H71/RF/B/02/27/016/002.

130 Kelley, *Surgical Diseases of Children*, 232.

131 Alice Newman, Mr Berry (Women Part II: June-December 1910), H71/RF/B/02/05/022/002.

132 Kelley, *Surgical Diseases of Children*, 49. For more on the language and the expression of pain in general, including in children, see Joanna Bourke, *The Story of Pain: From Prayer to Painkillers* (Oxford: Oxford University Press, 2014).

133 John Normile, Mr Dodd (Men and Women Part II: 1897-1903), H71/RF/B/02/13/001/002.

134 Albert Boakes, Mr Berry (Men Part III: June-December 1909), H71/RF/B/02/05/020/003.

135 Jalland has suggested that the early twentieth-century British upper class and the lower working class shared the tendency to display a stiff upper lip. See Pat Jalland, *Death in War and Peace: Loss and Grief in England, 1914-1970* (Oxford: Oxford University Press, 2010). For a discussion of different types of silence in the historical record in relation to children, see Pooley and Taylor, “Introduction,” in *Children’s Experiences*, 18-19.

136 Frederick Myers, Mr Roughton (Men Part I: January-June 1905), H71/RF/B/02/27/008/001.

137 Henry Padmore, Mr Roughton (Men and Women: 1904), H71/RF/B/02/27/007.

138 Walter Cutler, twice, Mr Roughton (Men Part II: 1901-1903), H71/RF/B/02/27/005/002.

139 William Hunter, Mr Roughton (Men Part I: December 1910-June 1911), H71/RF/B/02/27/020/001.

140 Richard Nash, Mr Berry (Men Part II: June-December 1900), H71/RF/B/02/05/002/002.

141 Catherine Willoughby, Mr Cunning (Women: January-December 1914), H71/RF/B/02/12/005.

142 Florence [Frances inside] Payne, Mr Berry (Women Part II: June-December 1904), H71/RF/B/02/05/011/002.

143 Campbell and Kerr, *Surgical Diseases of Children*, 5.

144 All are Mr Roughton’s patients: Mark Keen (Men Part II: April-December 1908), H71/RF/B/02/27/014/002; both Robert Buckingham and Edward Patrick (Men Part III: June-December 1910), H71/RF/B/02/27/018/003.

145 Harry Bracking, Mr Roughton (Men Part I: December 1910-June 1911), H71/RF/B/02/27/020/001; Lily F. Collins, Mr Berry (Women Part II: March-November 1909), H71/RF/B/02/05/021/002.

146 Cooter, *Surgery and Society*, 60.

147 For surgery causing problems for children, see Brock, “Surgery, Success and the Role of the Patient.”

148 See for example, Walter Pye, *The Surgical Treatment of the Common Deformities of Children* (Bristol: John Wright & Co.,1889), where “surgical treatment” is primarily advocated as mechanical.

